# Isolated condylar fractures diagnosed by post mortem computed tomography

**DOI:** 10.1007/s12024-019-00104-7

**Published:** 2019-03-12

**Authors:** Aleksandra Borowska-Solonynko, Victoria Prokopowicz, Dorota Samojłowicz, Małgorzata Brzozowska, Jarosław Żyłkowski, Leszek Lombarski

**Affiliations:** 1 Department of Forensic Medicine, Medical University of Warsaw, 1 Oczki st., 02-007 Warsaw, Poland; 2 Second Department of Clinical Radiology, Medical University of Warsaw, 1a Banacha st., 02-097 Warsaw, Poland; 3 Department of Neurosurgery, Medical University of Warsaw, 1a Banacha st., 02-097 Warsaw, Poland

**Keywords:** Postmortem computed tomography, Occipital condylar fractures, High-energy mechanical injuries, Death scene reconstruction

## Abstract

Due to their anatomical location, occipital condylar fractures (OCFs) are usually not observed during traditional autopsies and are therefore considered a rare injury. The aim of this study was to determine the true frequency of OCFs using post-mortem computed tomography (PMCT) in traumatic casualties. We retrospectively analyzed 438 PMCT studies of victims of traffic accidents, falls from height, violence, and low-energy head injuries (324 males and 114 females). OCFs were present in 22.6% of cases (*n* = 99), mostly in victims of railway accidents (48.5%, *n* = 17), falls from height (26.6%, *n* = 29), cyclists (24%, *n* = 6), and pedestrians hit by cars (22.5%, n = 29). Isolated OCFs were found in 5.5% of cases (*n* = 24), most often in cyclists (12%, *n* = 3) and pedestrians (9.3%, *n* = 12) hit by cars. There were no OCFs in the cases of fatalities caused by violence or accidental low-energy head injury. PMCT scans revealed that OCFs are common in high-energy injury fatalities and can be useful for determining the mechanism of trauma more precisely.

## Introduction

A large portion of the occipital condylar surface is not readily palpable from the side of the cranial cavity. In order for it to be exposed for a thorough examination, the occipital condyles must be separated from the first cervical vertebra, which is not routinely done during an autopsy. As a result, occipital condylar fractures (OCFs) are often not detected during traditional autopsies, and before the widespread use of postmortem imaging techniques, OCFs were considered rare injuries [1]. Although Leone et al. [2] claimed that OCFs should not be considered completely infrequent and may occur in as many as 16% of patients with craniocervical injury in 2000, the general belief that OCFs are uncommon persists. This conviction extends beyond forensic pathology and into clinical medicine [3–8], with the pediatric population considered to have the lowest incidence of OCFs [9, 10].

The first documented OCF was identified during an 1817 autopsy performed by Sir Charles Bell [11]. The autopsy was conducted on a male who had fallen from height. The man had fallen backwards off a wall but was only diagnosed with a slight concussion. However, when he turned his head and neck to bid farewell to the hospital staff who had taken care of him after the accident, he dropped dead. The autopsy revealed that when he had suddenly turned his head, the fractured condyle was displaced, and the loose bone compressed the medulla oblongata. After that event, the first radiographic evidence of an OCF in vivo was reported in 1962 [12], and the first computed tomographic (CT) scans of OCFs were published in 1983 [13, 14]. However, since 1817, fewer than two hundred cases of OCFs have been reported in medical literature [7].

The aim of this study was to use PMCT to determine the true frequency of OCFs in fatal injury cases.

## Materials and methods

PMCT scans were obtained using a 16-row Astelion CT scanner (Toshiba). In each of these cases we performed unenhanced CT scans, with 1-mm-thick slices acquired at 120 V with automatic exposure control (AEC). The pitch factor was 1.438 for the trunk and 0.688 for the head. The scans were analyzed by a forensic medicine specialist (13 years of expertise) and a radiologist (12 years of expertise) using OsiriX software (non-commercial license).

This study involved a retrospective evaluation of 438 PMCT examinations performed in the Department of Forensic Medicine at the Medical University of Warsaw between November 2014 and December 2016. The inclusion criterion was a traumatic death that had occurred in known circumstances.

The cases with PMCT-confirmed OCFs were divided into three subgroups according to the Anderson and Montesano classification system [15]: type I - an impaction-type fracture with occipital condyle comminution, type II – a linear fracture as part of a basioccipital fracture, and type III - an avulsion fracture. Types I and III indicate isolated OCFs, which means OCFs that are not directly connected to other fractures of the skull. From the medico-legal point of view, type II OCFs are less significant than isolated OCFs due to the presence of other extensive head injuries in type II OCFs; this is why an emphasis was put on analyzing isolated OCFs. Thus, apart from PMCT scans, we analyzed the respective autopsy reports in the cases of isolated OCF.

TIBCO Software Inc. Statistica (data analysis software system), version 13 (2017) was used for statistical analysis. Pearson’s chi-squared tests and Fisher’s exact test (due to the limited number of cases) were used to compare nonparametric, nominal variables such as: sex, the presence or absence of an OCF, the type of the OCF, the side of the OCF, lateralization of head injuries, and the mechanism of death. The following correlation coefficients were also used: coefficient Φ and the contingency coefficient C. The results were considered statistically significant when the adjusted *p* values were less than 0.05 (*p* < 0.05).

## Results

Victims of traffic accidents made up the largest number of cases (70%, *n* = 300), followed by falls from heights (24.8%, *n* = 109), violence, and accidental low-energy head injuries (5.2%, *n* = 23). The examined decedents included 324 males (74%) and 114 females (26%).

OCFs were present in 22.6% (*n* = 99) of the 438 evaluated PMCT examinations. Among all the OCFs (n = 99), the largest group (*n* = 75) was Anderson and Montesano type II, which constituted 75.6%, with type III fractures (*n* = 21) accounting for 21.1%. Only three (3.03%) type I OCFs were found. The relation between the presence of OCFs and sex was assessed (Table 1). OCFs were found in 27.19% of women (*n* = 31) and 21.45% of men (*n* = 68). This relation was not statistically significant (Pearson’s chi-squared tests *p* = 0.211). However, women were observed to have a larger percentage of isolated OCFs (35.48% of all OCFs found in women), than men (with isolated OCFs constituting only 19.12% (*n* = 13) of all OCFs found in men). The obtained result was not statistically significant (*p* = 0.067). The correlation between sex and the type of condylar fracture (isolated OCF vs. other OCF) was very low (Φ = −0.17 and C = 0.17).

**Table 1:** Occipital condylar fractures (OCFs) in relation to victim’s sex

Victim’s sex	OCF not present	OCF present		Final sum
		Other type OCF	Isolated OCF	
Femal	83 (72,8%)	20 (17,54%)	11 (9,65%)	114
Male	249 (78,5%)	55 (17,35%)	13 (4,1%)	317
Final sum	332	75	24	431

Subsequently, we assessed the relationship between the presence of OCF and the mechanism of death based on medico-legal data (Table 2). OCFs occurred most commonly in victims of railway accidents (48.5%, *n* = 17), falls from height (26.6%, *n* = 29), and in cyclists (24%, *n* = 6) and pedestrians (22.5%, n = 29) hit by cars. OCFs were not found in victims of violence or accidental low-energy head injuries. Analysis showed significant differences with respect to the proportion of OCF-positive decedents between the groups of different mechanisms of death (*p* = 0.001). However, the strength of the correlation between the mechanism of death and the presence of OCFs, was low (C = 0.23).

**Table 2:** Occipital condylar fractures (OCFs) in relation to victim’s mechanism of death

Mechanism of death	OCF not present	OCF present		Final sum
		Other type OCF	Isolated OCF	
Other	8 (100%)			8
Motor vehicle accident – driver	42 (85,71%)	5 (10.2%)	2 (4,08%)	49
Motor vehicle accident – motorcyclist	23 (76,7%)	6 (20%)	1 (3,33%)	30
Motor vehicle accident – passenger	28 (87,5%)	4 (12,5%)		32
Pedestrian hit by car	100 (77,52%)	17 (13,18%)	12 (9,3%)	129
Violences	14 (100%)			14
Cyclist hit by car	19 (76%)	3 (12%)	3 (12%)	25
Fall from height	80 (73,39%)	25 (22,93%)	4 (3,67%)	109
Railway accident	18 (51,43%)	15 (42,86%)	2 (5,71%)	35
Final sum	332	75	24	431

**Fig. 1:**
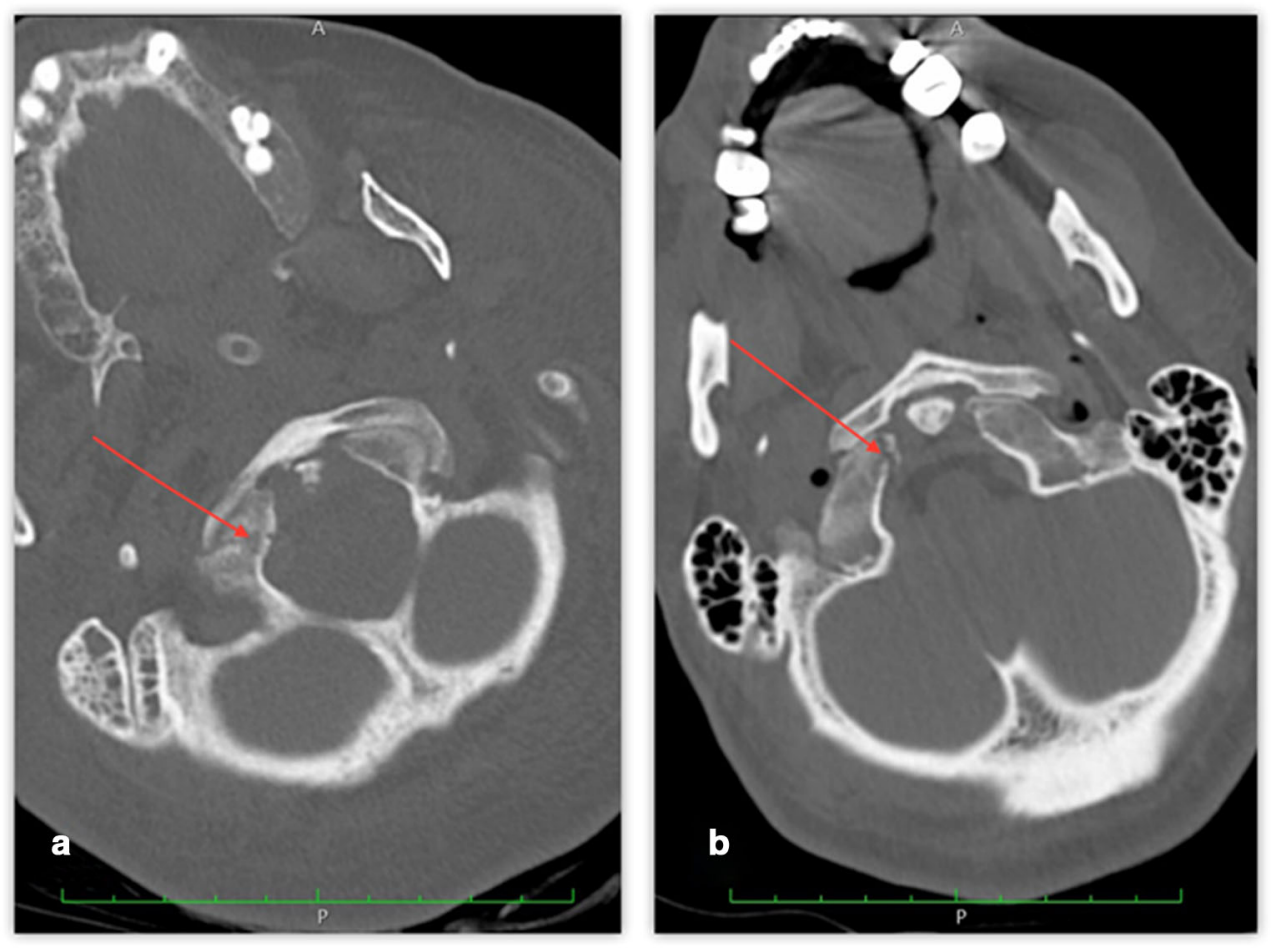
Postmortem computed tomography (PMCT) multiplanar reconstruction (MPR) images showing isolated occipital condylar fractures (OCFs). **a** – type I OCF, **b** – type III OCF

Specific analysis was performed on isolated OCFs (Anderson and Montesano types I or III (Fig. 1.)). Isolated OCFs were found in 5.5% (*n* = 24) of the 438 PMCT cases. Due to the small number of isolated fractures, Anderson-Montesano type I and III OCFs were analyzed together.

**Fig. 2:**
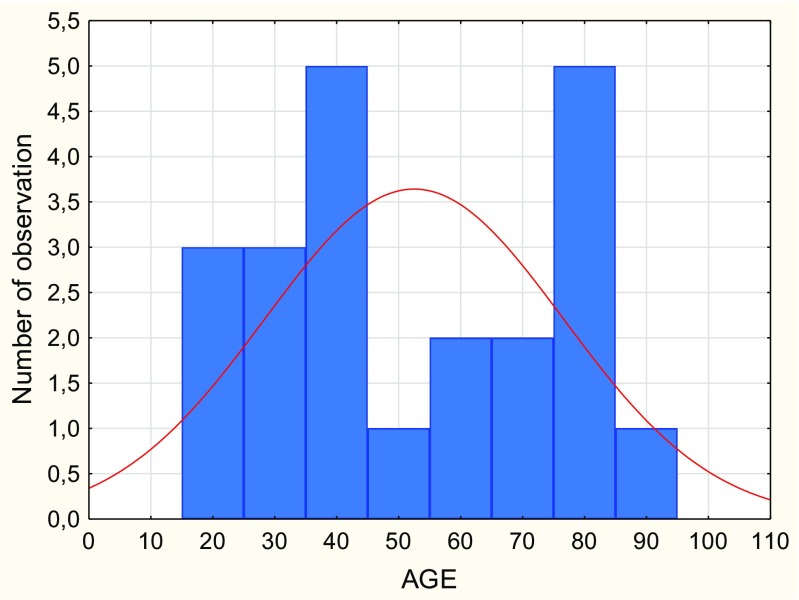
Age distribution of people with isolated occipital condylar fractures (OCFs)

Isolated OCFs were found in every age group (Fig. 2). The youngest person with this injury was 16 years old, the oldest 95 years old. The median age was 51.65 years.

The largest proportions of isolated OCFs were observed in cyclists and pedestrians, at 50% (*n* = 3) and 41.38% (*n* = 12) of all OCFs in these respective groups. In 7 out of the 12 pedestrian cases (58.3%), the location of lower extremity injuries (including crushing and de-gloving injuries, as well as the presence of torn cruciate ligaments) indicated that these people had been in a vertical position, with either their front or back facing the oncoming vehicle at the moment of impact. In 3 cases (25%), the pedestrians had a lateral surface of their body facing the oncoming vehicle. In two cases it was not possible to determine the position of the body at the moment of impact. One of these two pedestrians died 1.5 months after the accident in question. Since the number of pedestrian cases with isolated OCFs was small (*n* = 12), it was not possible to draw any statistically sound conclusions regarding any relationship between the presence of an isolated OCF and the position of the pedestrian at the time of the collision. The presence of isolated OCFs was only confirmed in two drivers, one of whom had not had his seatbelt buckled at the time of the accident. No information was available about the use of seatbelts by the other driver. According to the data obtained from the prosecutor’s office, the one case of a motorcyclist with an isolated OCF involved him having driven directly into the back of a semi-trailer.

Out of the total number of cases with isolated OCFs, the cause of death was mainly multi-organ trauma (74%, *n* = 17). In 8.7% (*n* = 2) of cases the cause of death was not determined because a traditional autopsy had not been performed. Other causes of death (17.3%, *n* = 5) included head trauma, head and cervical spine injury, pulmonary embolism (in a person in poor neurological condition due to head injury, hemorrhagic shock due to traumatic amputation of the lower leg, and heart failure (in an 80-year-old woman who died after a 1.5-month hospitalization having sustained injuries to multiple locations on the body. In all of the isolated-OCF cases in which an autopsy was performed, other findings which were sufficient to explain the cause of death were revealed.

Traditional autopsies involving victims with isolated OCFs showed the presence of facial skin injuries in the majority of cases (90.5%, *n* = 19). In the two cases in which facial skin injuries were not found, each of the relevant autopsies involved a pedestrian hit by a car and hospitalized for several (3 and 6) weeks after the accident. Fractures of the base of the skull were not observed in 87% (*n* = 20) of the cases with an isolated OCF. Fractures of the base of the skull were found in the remaining cases; however, the fracture lines did not extend into the occipital condylar region. Fractures of the cervical spine were reported to be absent in 65% (*n* = 15) of traditional autopsies. In those cases forensic pathologists did not describe any injuries in the craniocervical junction even though such injuries were in fact present in the form of isolated OCFs. In 23.8% of cases (*n* = 5), the autopsy found damage to the spinal cord in the form of tearing or severance (*n* = 4), as well as hemorrhages beneath the pia mater (*n* = 1).

Most of the isolated OCFs were left unilateral fractures (66.66%, *n* = 16), followed by right unilateral (31%, *n* = 7) and bilateral (4%, n = 1) fractures. Based on the description of soft tissue injuries and bone fractures in the individual autopsy reports, side lateralization of these head injuries was determined. Table 3 shows the relationship between the side of the isolated OCF and the side on which the majority of head injuries were present. We observed that OCFs were usually found on the opposite side to the head injury, however, the difference between the side of the OCF and the side lateralization of head injuries was not statistically significant (*p* = 0.08). This could be a result of the number of evaluated cases being too low. Further statistical analysis showed a significant correlation between the above groups (C = 0.52).

**Table 3:** Side of isolated occipital condylar fractures (OCFs) in relation to the lateralization of head injuries

Isolated OCF	Dominant head injuries			Final sum
	Left	Both sides	Right	
Left	5 (55,66%)	2 (66,67%)	7 (77,78%)	14
Both sides		1 (33,33%)		1
Right	4 (44,44%)		2 (22,22%)	6
Final sum	9	3	9	21

## Discussion

The present study demonstrated that OCFs of all Anderson and Montesano types occur quite frequently; in as many as 22.6% of victims of traumatic deaths; with the highest rates seen in deaths caused by high-energy mechanical injuries, especially railway accidents. This is consistent with the conclusion by Hanson et al. [16] that OCFs are markers of high-energy trauma [16]. In the examined material, there were no cases in which an OCF was found in victims of violence, which may be important information in certain cases, such as ones in which there is a need to distinguish the effects of beatings from those of traffic accidents. Looking at Anderson and Montesano’s classification [13], which we chose to categorize OCF types, it is justified to conclude that the character of an OCF may more precisely determine the mechanism of trauma. Type I fractures arise as a result of axial forces leading to the fragmentation of the condyle. Type II fractures result from cranial base fractures which continue onto the occipital condyles. Type III fractures are avulsion fractures associated with trauma affecting the alar ligaments [1, 7, 17]. Stretching forces along either of these ligaments cause the ipsilateral condyle to fracture, at the site of ligament attachment, but the contralateral ligament can also be damaged. The function of the alar ligament is to prevent excessive rotation of the odontoid process of the axis [4]. Therefore, it follows that a type III OCF indicates excessive rotation of the head. This is consistent with our observations of the frequent occurrence of OCFs contralateral to the side of the head with most pronounced injuries. In a short article describing two cases of victims of traffic accidents, Hansen et al. [17] put forward the hypothesis that, in addition to head rotation, the body of the victim must simultaneously move forward for an OCF to occur. Taking into account the types of injuries in which OCFs were observed, both in our study and in many others, it should be noted that in almost all cases the victim had been moving rapidly forward at the moment of impact. This rapid forward movement may also explain the possibility of OCF formation under the influence of slightly weaker forces as described in a paper by Kapapa et al. [9]. That paper reported an OCF diagnosed in a child who fell from a carousel. Our detailed analysis of the injuries to those pedestrians who were confirmed to have isolated OCFs showed that these types of fractures were associated with an impact to the front or back of the body, which means that not only a sudden movement forward but also backward can influence the occurrence of OCFs. Of course, these movements would end with a rotation of the head (e.g. as a result of a collision with parts of the vehicle). Proving that type III OCFs correlate with a rapid forward flexion of the body may potentially have large significance in establishing whether or not the victim had fastened her/his seatbelts; however, this hypothesis requires further investigation. In our research isolated OCFs were found in two drivers, one of whom definitively did not have his seatbelts fastened. An OCF may also indicate an incorrect position of a passenger or driver in the event of airbags opening, as stated in the case report by Maxeiner et al. [18]. Tuli et al.’s [19] classification, which is based on the stability of the O-C1-C2 joint complex, is not useful for determining the mechanism of trauma, as it is noted in the literature that this classification does not provide precise definitions [20].

OCFs are often overlooked in both clinical and autopsy settings. According to the currently available medical literature, CT scans are considered superior to conventional radiography [3, 20–22] when it comes to diagnosing OCFs. The widespread use of CT in clinical practice has caused this injury (once considered rare and occurring in less than 1% of traumatic cases [3]), to be detected more often in patients hospitalized after trauma. Furthermore, recently there have been an increasing number of clinical reports in which attention is paid not only to the existence of this type of injury, but also to the complications that develop in patients with an OCF. These complications include brachial plexus palsy [23], cranial nerve palsy [24], or vascular complications such as blunt traumatic vertebral artery injury [25].

The topic of OCFs is virtually ignored by forensic pathologists, apart from a few isolated publications [1, 17]. This can be explained by the fact that, due to their location, occipital condyles are practically never examined during traditional autopsies. Occipital condyles are largely inaccessible during traditional autopsy without employing special additional techniques, such as separating the base of the skull from the first cervical vertebra. Palpation from the cranial cavity only allows access to the medial surface of the condyles; however, due to the presence of ligaments stabilizing the craniovertebral junction, even the fractures involving the medial condylar surface can be difficult to palpate if they are not displaced. Our research demonstrated that the majority of isolated OCFs were not accompanied by any other fractures of the base of the skull or by detectable cervical spine injuries. In these cases, forensic pathologists were mistakenly under the impression that the craniocervical transition region sustained no injuries, even though it actually had. This confirmed the hypothesis raised by some authors that the rates of damage not only to the condyles themselves but also to the upper portion of the cervical spine are severely underestimated [25]. It is only by complementing the conventional post-mortem examination with PMCT that occipital condyle assessment becomes fairly simple. Even though PMCT is used increasingly in medico-legal practice, it is unfortunately still not common. Therefore, the importance of OCFs from the medical and legal point of view is, for the moment, unknown. Our research did not provide evidence that OCFs alone are a cause of death in the cases that we studied. However, the results or our study as well as data from the literature indicate that confirming the presence of OCFs, especially isolated OCFs, may be useful in reconstructing the mechanism of injury. The results of our research and previously published literature show that PMCT plays an important role in complementing the traditional autopsy, particularly in situations of suspected craniocervical-junction or cervical-spine injuries [26, 27]. The authors of this study believe the topic of OCFs in forensic medicine, together with the increasing use of PMCT, will be more and more readily studied.

## Key points

1. PMCT revealed that OCFs are quite common in deaths caused by high-energy mechanical injuries.
2. OCFs can occur both with and without additional craniocervical injuries and, therefore, should always be sought in cases of high-energy trauma.
3. Isolated OCFs are most likely occur as a result of violent head rotation combined with lateral bending and are more common in women than in men.
4. The diagnosis of OCFs, especially isolated OCFs, may be useful for death scene reconstruction.
